# Vacuum ultraviolet photodissociation of sulfur dioxide and its implications for oxygen production in the early Earth's atmosphere†

**DOI:** 10.1039/d3sc03328g

**Published:** 2023-08-01

**Authors:** Yao Chang, Yanlin Fu, Zhichao Chen, Zijie Luo, Yarui Zhao, Zhenxing Li, Weiqing Zhang, Guorong Wu, Bina Fu, Dong H. Zhang, Michael N. R. Ashfold, Xueming Yang, Kaijun Yuan

**Affiliations:** a State Key Laboratory of Molecular Reaction Dynamics, Dalian Coherent Light Source, Dalian Institute of Chemical Physics, Chinese Academy of Sciences 457 Zhongshan Road Dalian 116023 China kjyuan@dicp.ac.cn bina@dicp.ac.cn xmyang@dicp.ac.cn; b Marine Engineering College, Dalian Maritime University Liaoning 116026 China; c School of Chemistry, University of Bristol Bristol BS8 1TS UK; d University of Chinese Academy of Sciences Beijing 100049 P. R. China; e Hefei National Laboratory Hefei 230088 China; f Department of Chemistry, Center for Advanced Light Source Research, College of Science, Southern University of Science and Technology Shenzhen 518055 China

## Abstract

The emergence of molecular oxygen (O_2_) in the Earth's primitive atmosphere is an issue of major interest. Although the biological processes leading to its accumulation in the Earth's atmosphere are well understood, its abiotic source is still not fully established. Here, we report a new direct dissociation channel yielding S(^1^D) + O_2_(a^1^Δ_g_/X^3^Σ_g_^−^) products from vacuum ultraviolet (VUV) photodissociation of SO_2_ in the wavelength range between 120 and 160 nm. Experimental results show O_2_ production to be an important channel from SO_2_ VUV photodissociation, with a branching ratio of 30 ± 5% at the H Lyman-α wavelength (121.6 nm). The relatively large amounts of SO_2_ emitted from volcanic eruptions in the Earth's late Archaean eon imply that VUV photodissociation of SO_2_ could have provided a crucial additional source term in the O_2_ budget in the Earth's primitive atmosphere. The results could also have implications for abiotic oxygen formation on other planets with atmospheres rich in volcanically outgassed SO_2_.

## Abstract 2

The emergence of molecular oxygen (O_2_) in the Earth's primitive atmosphere is an issue of major interest.

## Graphical abstract

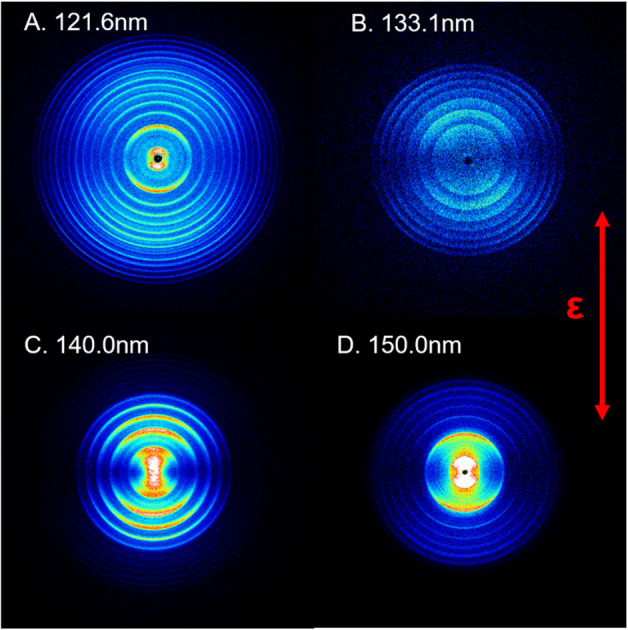

## Introduction

The provenance of oxygen (O_2_) remains a crucial topic in the history of the Earth's evolution. The Earth's present atmosphere is notable for its remarkably high concentration of O_2_ (∼21% by volume), whereas geological and geochemical constraints suggest that free oxygen was anything but plentiful during the first half of the Earth's 4.5 billion year history.^1–3^ A permanent rise to appreciable concentrations of O_2_ in the atmosphere, known as the “Great Oxidation Event (GOE)”,^4–8^ is estimated to have occurred ∼2.4 billion years ago (2.4 Ga). The GOE could have been a consequence of the emergence of oxygenic photosynthesis. Alternatively, O_2_ biogenesis may be much older; several pieces of evidence point to the first emergence of oxygenic photosynthesis long before the GOE (as early as ∼3.8 Ga).^9–14^ If so, the emergence of the GOE could have been a consequence of an abiotic shift in the balance of oxidants and reductants at the Earth's surface, *i.e.*, at early times, the biogenically produced O_2_ was effectively consumed *via* reaction with reduced compounds, thereby suppressing O_2_ levels (<10^−6^ of the present atmospheric level (PAL))^15–17^ but, eventually, this source-sink balance shifted in favour of O_2_ accumulation. Recent trace metal studies, *e.g.* of molybdenum and rhenium enrichment in the crust,^10,18^ suggested “whiffs” of O_2_ in the late Archaean (2.5–2.7 Ga), *i.e.*, intermittent periods before the GOE when the O_2_-sources overwhelmed the sinks. Such whiffs of oxygen have been suggested as a possible trigger for the GOE, but their duration, magnitude, and sources remain unknown.

Apart from biogenic processes leading to O_2_ production, the widely accepted abiotic route to forming O_2_ is the three-body recombination reaction O + O + M → O_2_ + M, involving O atoms produced by photolysis of CO_2_ or other oxygen-containing molecules.^19–22^ Direct O_2_ production *via* vacuum ultraviolet (VUV) photodissociation of CO_2_ has also been identified, but deduced to be a very minor process compared with the indirect three-body recombination.^23^ Dissociative electron attachment to CO_2_ has also been shown to lead to direct O_2_ production.^24^ Haqq-Misra *et al.*^25^ have suggested another abiotic O_2_ production pathway, in which H_2_O_2_ was first produced from the by-products of H_2_O photolysis, and then converted to O_2_*via* disproportionation reactions. Modelled O_2_ concentrations up to 10^−7^ PAL were reported. These findings have all informed knowledge of the history of the Earth's atmosphere and our understanding of planetary atmospheres and of interstellar photochemical processes.

SO_2_ has also been considered as a possible source of oxygen. Astronomical observations have identified high SO_2_ concentrations in the atmosphere of terrestrial exoplanets like Venus^26,27^ and Io,^28,29^ which are largely attributable to outgassing from volcanoes. In the Earth's late Archaean, prior to the GOE, subaerial volcanic degassing became important, yielding gases much richer in sulfur and dominated by SO_2_.^30,31^ The photochemistry of volcanic SO_2_ has been studied extensively in recent decades and has commonly been linked to the origin of the sulfur mass independent fractionation (S-MIF) in ancient rock samples.^32–34^ SO_2_ should be the major component from volcano eruptions, but the role of SO_2_ photochemistry in the formation of molecular oxygen in the Earth's early atmosphere has hitherto been largely ignored.

SO_2_ has two strong absorption bands in the UV region (see Section S2 and Fig. S2 in the ESI†). The more intense band, assigned to excitation from the ground X̃^1^A_1_(1^1^A′ in *C*_s_) state to the C̃^1^B_2_(2^1^A′) state, spans the wavelength range 185 < *λ* < 235 nm.^35^ The other strong band at longer wavelengths, *λ* ∼ 240–350 nm, is associated with transition to the B̃^1^B_1_(2^1^A′′) state. The B̃^1^B_1_(2^1^A′′) state potential energy surface displays a conical intersection with that of the first excited singlet state (the Ã^1^A_2_(1^1^A′′) state).^33,36,37^ Weak absorption at yet longer wavelengths is attributed to the spin-forbidden transition to the ã^3^B_1_ state. Photoexcitation of SO_2_ at wavelengths *λ* ≤ 219 nm results in predissociation, primarily to SO and O fragments, driven by non-adiabatic couplings to the lower-lying dissociative singlet and triplet states.^38–41^ As Fig. S2† shows, the SO_2_ absorption spectrum at shorter (VUV) wavelengths displays several intense diffuse absorption bands assigned to transitions to Rydberg states.

Early investigations of the VUV photolysis of SO_2_ found indirect experimental evidence for the S + O_2_ channel through detection of OH fluorescence from SO_2_/H_2_ mixtures.^42^ More recently, Rosch *et al.*^43^ reported the first direct evidence for the S(^3^P) + O_2_ channel from SO_2_ photolysis at 193 nm but, in the absence of quantitative measurements, the assessment of the importance (or otherwise) of O_2_ production from VUV photodissociation of SO_2_ in the Earth's primitive atmosphere was not possible. The recent development of the intense VUV free electron laser (FEL) at the Dalian Coherent Light Source (DCLS) provides a unique tool for studying molecular photofragmentation dynamics across the entire VUV range.^22,44,45^ Here, we present careful experimental studies of the S(^1^D) + O_2_ product channel following SO_2_ photolysis at various wavelengths in the range 120 < *λ* < 160 nm using the VUV-pump and VUV-probe time-sliced velocity-map imaging (TS-VMI) technique, along with complementary electronic structure calculations. The quantitative assessment of this channel suggests that the VUV photochemistry of SO_2_ could have been an important additional source of O_2_ in the Earth's atmosphere in the late Archaean.

## Results and discussion

### The verification of the S(^1^D) + O_2_ product channel

In this study, the photodissociation dynamics of SO_2_ have been investigated using the recently constructed VUV pump and VUV probe TS-VMI apparatus, which is equipped with two independently tunable VUV laser radiation sources (see Section S1 and Fig. S1 in the ESI†). A pulsed supersonic molecular beam generated from a gas mixture of about 1% SO_2_ in Ar was irradiated with two counter-propagating VUV beams. The VUV FEL output was used to excite SO_2_ molecules to different Rydberg states at wavelengths in the range 120 < *λ* < 160 nm. The S(^1^D) photofragments were then resonantly ionized with *λ* = 130.092 nm photons, which were generated using a table-top VUV source and a difference frequency four-wave mixing (FWM) scheme, involving two 212.556 nm photons and one 580.654 nm photon that were overlapped in a Kr gas cell. Post-ionization, the S(^1^D) photoproducts were detected by the high resolution VMI detector.

**Fig. 1:**
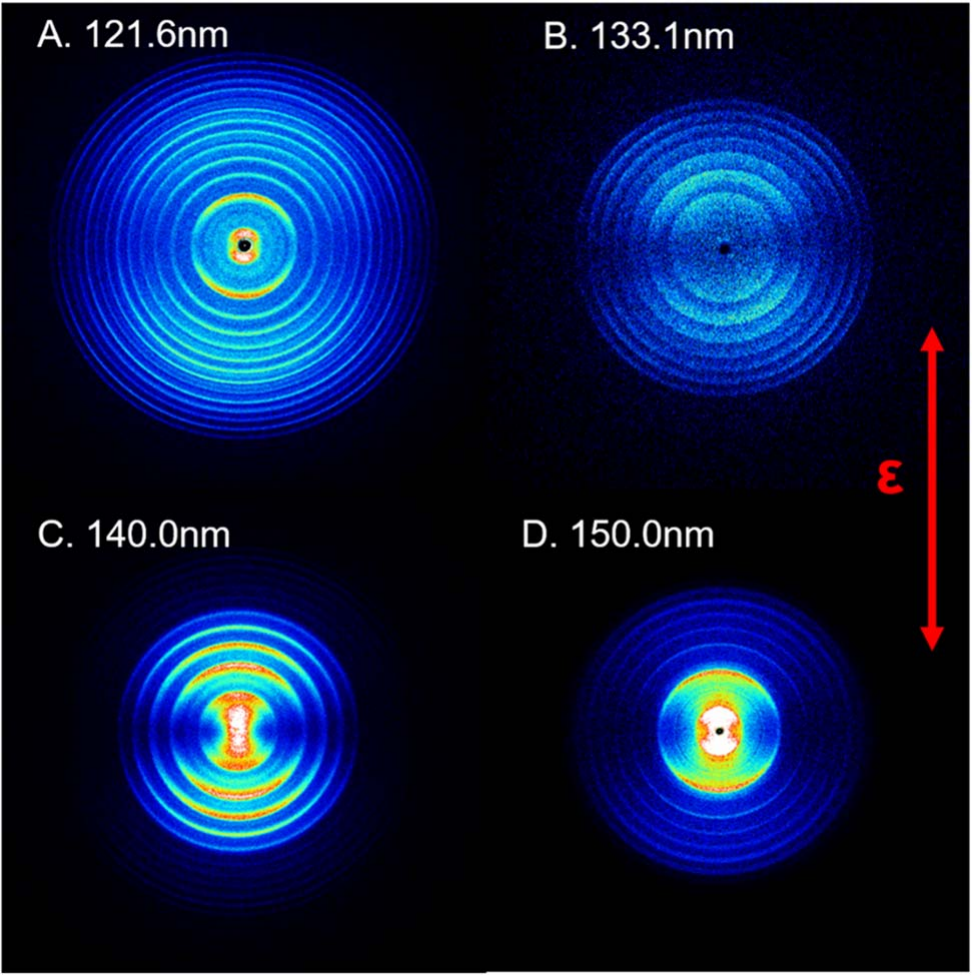
Time-sliced images of the S(^1^D) products from SO_2_ photodissociation. The images were recorded at photolysis wavelengths *λ* = (A) 121.6 nm, (B) 133.1 nm, (C) 140.0 nm and (D) 150.0 nm. The double headed red arrow indicates the polarization direction of the photodissociation laser, *ε*. The concentric ring features reflect the population of different vibrational levels of the coincident O_2_(X^3^Σ_g_^−^/a^1^Δ_g_, *v*) products.

Fig. 1 shows time-sliced ion images of the S(^1^D) photofragments recorded following photolysis of SO_2_ at VUV wavelengths *λ* = 121.6, 133.1, 140.0 and 150.0 nm, respectively. Additional images, taken at *λ* = 125.1, 130.1, 144.1 and 154.1 nm, are displayed in Fig. S3 of the ESI.† The double headed arrow in Fig. 1 shows the direction of the polarization vector of the photolysis laser. Well-resolved, concentric rings with different intensities are clearly observable in the displayed images. These structures can be readily assigned to the population of different vibrational levels of the O_2_ co-product in its ground (X^3^Σ_g_^−^) or first excited (a^1^Δ_g_) electronic state arising *via* the photodissociation channel (1),

1 SO_2_ + *hv* → S(^1^D) + O_2_.

The triple dissociation channel yielding S(^1^D) + O + O products has a threshold energy of ∼12.3 eV^46^ and is thus not accessible in the present experiment. The use of an off-axis biconvex LiF lens as the exit window for the four-wave mixing cell ensured that the 212.556 nm and 580.654 nm laser beams were dispersed from the photodissociation/photoionization region, thereby eliminating the possibility of unintended secondary dissociation of any primary SO fragments (from the rival SO + O dissociation channel) by absorption of another UV or IR photon. In addition, both the VUV FEL beam and the 130.092 nm probe beam were kept defocused to minimize any two-photon excitation effects. These steps ensured that no processes other than channel (1) yielded S(^1^D) fragments under the prevailing experimental conditions.

**Fig. 2:**
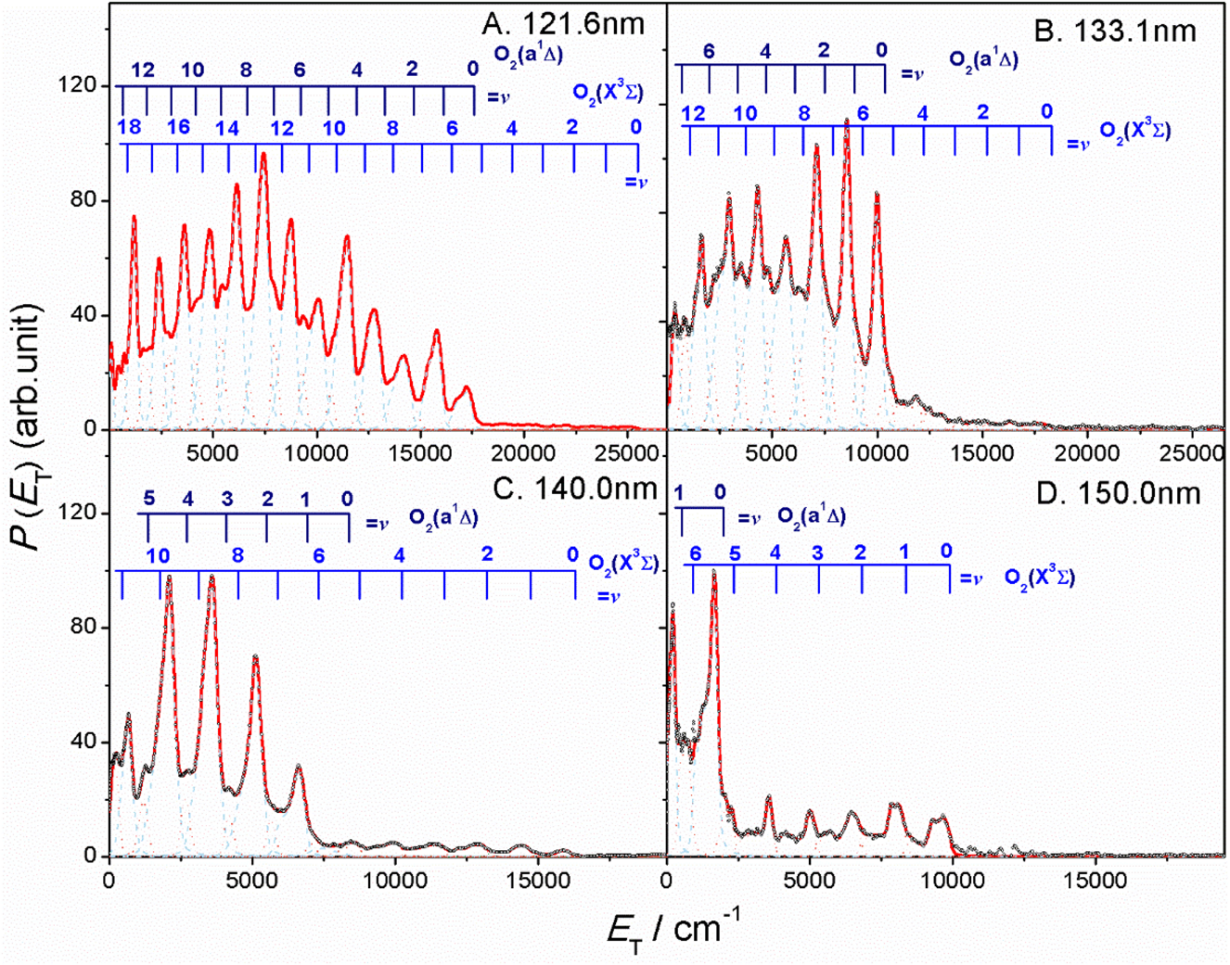
The product total kinetic energy (*P*(*E*_T_)) spectra for S(^1^D) + O_2_ products following photolysis at *λ* = (A) 121.6 nm, (B) 133.1 nm, (C) 140.0 nm and (D) 150.0 nm, derived from the images shown in Fig. 1, in red, along with the best-fit simulation of the spectra, in cyan dashed lines and orange dotted lines. The superposed combs indicate the *E*_T_ values associated with the formation of the various vibrational levels of O_2_(X^3^Σ_g_^−^/a^1^Δ_g_, *v*).

The thermochemical threshold for process (1), *i.e. D*_0_[SO_2_(X̃, *v* = 0) → S(^1^D) + O_2_(X, *v* = 0)], is ∼7.1 eV (corresponding to an excitation wavelength, *λ* ∼ 175 nm).^46^ Any energy provided by the VUV photon (*E*_*hv*_) in excess of this threshold energy will be deposited into the fragment kinetic energy and/or into the internal energy (*E*_int_) of the O_2_ products. The radii of the well resolved ring structures in the TS-VMI images can be used to determine the velocity distribution of the S(^1^D) products. These velocities can then be converted to a total kinetic energy release *P*(*E*_T_) spectrum of the S(^1^D) + O_2_ products using linear momentum conservation arguments. Fig. 2 and S4 (in the ESI†) display the *P*(*E*_T_) spectra of the S(^1^D) + O_2_ products obtained by integrating signals over all angles in the respective images. The internal energy distributions of the O_2_ co-products, *E*_int_[O_2_] formed at each wavelength can then be obtained from the corresponding *P*(*E*_T_) spectrum using the law of energy conservation (eqn (2)).

**Fig. 3:**
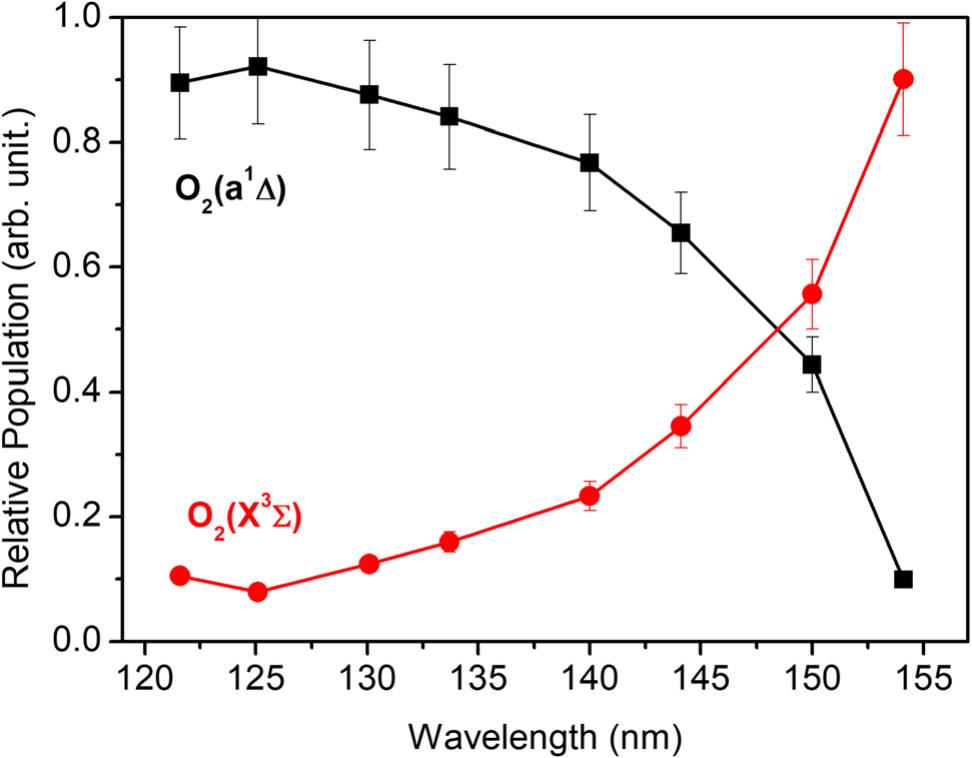
The relative population of the O_2_(a^1^Δ_g_) and O_2_(X^3^Σ_g_^−^) products as a function of photolysis wavelength.

2 *E*_*hv*_ − *D*_0_ = *E*_int_[O_2_] + *E*_T_[S(^1^D) + O_2_],

Each panel in Fig. 2 reveals two vibrational progressions for the O_2_ co-products, which can be assigned to the population of different vibrational levels of the ground (X^3^Σ_g_^−^) and first excited electronic (a^1^Δ_g_) state (the zero-point level of which lies 7882 cm^−1^ above that of the ground state^46^). The onset of the strong progression in each *P*(*E*_T_) spectrum accords well with the threshold of the S(^1^D) + O_2_(a^1^Δ_g_) product channel, providing unambiguous evidence for the formation of molecular O_2_ in the VUV photodissociation of SO_2_. Fig. 3 shows the relative populations of the O_2_ products formed in the two electronic states following SO_2_ photolysis at each wavelength studied, obtained from simulation of the *P*(*E*_T_) spectra (Fig. 2 and S4†). Clearly, the S(^1^D) + O_2_(X^3^Σ_g_^−^) channel is more important at the longest wavelengths investigated, but the S(^1^D) + O_2_(a^1^Δ_g_) channel becomes increasingly dominant upon tuning to shorter wavelengths. The O_2_(a^1^Δ_g_) and O_2_(X^3^Σ_g_^−^) fragments formed at all but the very longest wavelengths both display inverted vibrational state population distributions, spanning a wide range of vibrational levels (Fig. S5 and S6 in the ESI†). The finite bandwidth of the FEL source precludes detailed discussion of the rotational energy disposal in the O_2_ products from the observed vibrational peak profiles.

**Fig. 4:**
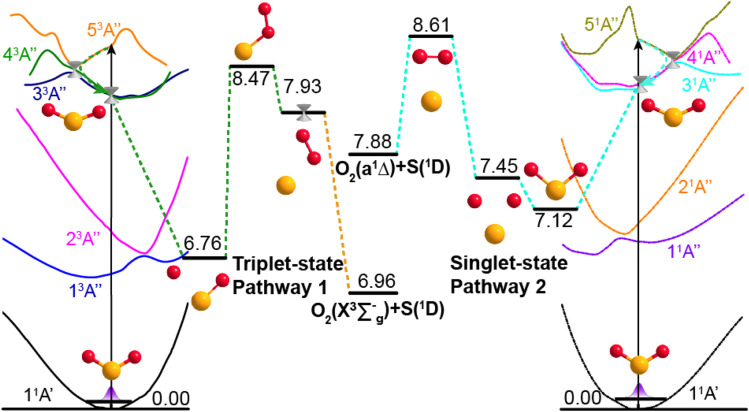
Dissociation pathways of SO_2_ leading to O_2_(X^3^Σ_g_^−^) + S(^1^D) products *via* the triplet state manifold (left) and to O_2_(a^1^Δ_g_) + S(^1^D) products *via* the singlet state manifold. The schematics of the simplified PECs for the ground and the first five excited states of ^3^A′′ symmetry (left) and the first five states of ^1^A′′ symmetry (right) support different paths leading to S + O_2_ product formation. Dashed arrows indicate possible non-adiabatic pathways and the geometries and energies (in eV) of key intermediate structures along the dissociation pathways are also indicated. The horizontal axis represents the reaction coordinate, and the vertical axis is the potential energy (in eV).

Potential energy surface (PES) calculations were also performed to gain insights into possible dissociation mechanisms for SO_2_ molecules excited to low lying Rydberg states. Fig. 4 depicts two possible dissociation pathways. The left half of Fig. 4 illustrates a triplet-state pathway, in which SO_2_ is initially excited to the 5^3^A′′ Rydberg state, and then undergoes non-adiabatic coupling to the 4^3^A′′ PES. The SO_2_ molecule evolves on the 4^3^A′′ state PES, and one O-atom roams away from the SO partner and then returns to abstract the other O-atom. This mechanism involves an S–O–O intermediate and eventual dissociation to S(^1^D) + O_2_(X^3^Σ_g_^−^) products on the 5^3^A′′ PES. The right half of Fig. 4 illustrates a dissociation pathway *via* the singlet-state manifold. Initial photoexcitation in this case is to the 5^1^A′′ state, which is followed by non-adiabatic coupling to the 3^1^A′′ PES and dissociation to form S(^1^D) + O_2_(a^1^Δ_g_) products. In this pathway, the interbond angle reduces, the two oxygen atoms approach towards a cyclic-SO_2_ intermediate and the O_2_ molecule is ejected. The identification of these pathways supports the experimental observation of O_2_(X^3^Σ_g_^−^) or O_2_(a^1^Δ_g_) products following VUV photodissociation of SO_2_. More details of the theoretical calculations are provided in Section S5 and Fig. S9 of the ESI.†

### The quantum yield of S(^1^D) + O_2_ products

To quantify the importance of O_2_ production from SO_2_ photolysis in the Earth's primitive atmosphere, we have sought to determine the branching ratios of all active dissociation channels. Since the H Lyman-α wavelength (121.6 nm) is most abundant in stellar VUV radiation and about 80% of all SO_2_ photodissociation events occur around the Lyman-α line,^47^ we have taken this as the representative wavelength to measure the branching ratios. Three fragment channels O(^1^D) + SO, O(^1^S) + SO and S(^1^D) + O_2_ formed by 121.6 nm photolysis have been detected using the VUV pump and VUV probe technique (Section S4 and Fig. S7 in the ESI†). Attempts to detect O(^3^P), S(^3^P) and S(^1^S) products under similar experimental conditions yielded no observable signals, suggesting that branching into each of the O(^3^P) + SO, S(^3^P) + O_2_ and S(^1^S) + O_2_ channels is negligible at this wavelength. By calibrating the detection efficiencies for the O(^1^D), O(^1^S) and S(^1^D) fragments (Section S4 and Fig. S8 in the ESI†), the branching ratio of the S(^1^D) + O_2_ product channel was determined to be ∼30 ± 5% at *λ* = 121.6 nm (as a fraction of all the detectable channels).

### Insights into the O_2_ budget in the ancient atmosphere

The present experimental results imply that O_2_ production is a significant process following VUV excitation of SO_2_. This finding could have profound implications for understanding the evolution of O_2_ in the Earth's primitive atmosphere. Current scenarios assume that biogenic O_2_ production started long before the GOE,^10–14^ but that any potential accumulation of O_2_ was offset by its consumption in reactions with reduced compounds emanating from the Earth's interior, *i.e.* O_2_ consumption balanced its production at these early times. This source-sink balance then shifted in favour of O_2_ accumulation, and finally triggered the GOE. This critical shift, relying on appreciable O_2_ production, is poorly understood. Geological studies suggest that the composition of the primitive atmosphere was probably determined by outgassing, through volcanism, and that the redox state of volcanic gases differs markedly between subaerial and submarine eruptions.^30,31,48^ Volcanic gases that erupted subaerially have generally equilibrated at high temperatures and low pressures with magmas close to the fayalite–magnetite–quartz buffer. Consequently, subaerial volcanism emitted SO_2_-dominated gas into the primitive atmosphere. Volcanic SO_2_ gas could reach the stratosphere during eruptions, whereupon photodissociation would contribute an additional O_2_ source. Such a scenario would introduce additional O_2_ sources that, temporarily, would exceed the available sinks.

Here we attempt to estimate the total O_2_ production from the VUV photochemistry of volcanic SO_2_ in the late Archaean. We start by assuming that the average SO_2_ emission per year from volcanoes in the late Archaean was the same as the modern volcanic outgassing rate on Earth (∼2.3 × 10^13^ gram per year (g per year)).^49^ When the volcanism subsided, SO_2_ was rapidly removed from the atmosphere by continued photolysis, gas-phase reactions and rain-out. Given that O_2_ production from SO_2_ photochemistry has here been shown to be a prominent pathway, we attempt to quantify the possible contribution of SO_2_ by assuming that VUV photodissociation coverts 1% of the total emitted SO_2_ directly into O_2_. The accumulated amount of O_2_ in the atmosphere from VUV photodissociation of SO_2_ during the late Archaean eon (∼200 million years) can then be estimated as follows,

3 *W*_O2_ = 2.3 × 10^13^ g per year × 200 × 10^6^ year × 1% = 4.6 × 10^19^ g

The total mass of O_2_ in the current Earth's atmosphere is ∼1.07 × 10^21^ g. Based on the above assumptions, the summed O_2_ from SO_2_ photolysis (eqn (3)) could be ∼4.3% of the present level of atmospheric O_2_ and this estimate should probably be viewed as a lower limit as it is likely that SO_2_ emissions and the UV flux were both higher in the late Archaean. Such arguments imply that O_2_ production from volcanic SO_2_ photochemistry could have provided substantial (and probably sporadic) contributions to the atmospheric O_2_ budget, given prevailing assumptions that most of the O_2_ sinks were already balanced by biogenic O_2_ production.^10–14^

We note that volcanic SO_2_ clouds and SO_2_-based aerosols typically only survive in the atmosphere for a short time (several weeks to a few years) before sinking, and that most incident VUV excitation of SO_2_ would likely occur in the stratosphere during the volcanic eruptions. (The incipient O_3_ column in the late Archean is transparent to VUV radiation and the CO_2_ column is also transparent to VUV photons around 121.6 nm.^50^) Within the average lifetime of SO_2_ in the atmosphere, the transient accumulation of O_2_ from SO_2_ photolysis can be estimated as ∼10^18^ to 10^19^ molecules per cm^2^ or ∼10^−5^ to 10^−6^ PAL. (This range was estimated assuming (i) a photon flux at *λ* ∼ 121.6 nm of ∼2 × 10^12^ photons per cm^2^ per s,^47^ (ii) that all VUV photons can be absorbed by SO_2_ clouds during the volcanic eruption and (iii) atmospheric lifetimes of SO_2_ between one month and one year). Such events could have led to short-lived “oxygen oases”, *i.e.*, localized or regional areas with significantly elevated O_2_ during the volcanic eruption. The photochemical activity of SO_2_ gas at 2.5–2.7 Ga has been linked with the abrupt rise of S-MIF signatures in sedimentary rocks around this time.^1,51,52^ Spikes in Mo, Se, and Re concentrations at 2.5 to 2.66 Ga, and their isotopic excursions, have also recently been interpreted in terms of transient sources, or “whiffs”, of O_2_.^10,18,53,54^ The present findings are consistent with these scenarios. We propose that the VUV photochemistry of volcanic SO_2_ can lead to efficient production of molecular O_2_ and could have led to transient and localised accumulations of O_2_ in the atmosphere before the GOE. Such transient elevations of O_2_ from SO_2_ photochemistry merit further consideration as a possible trigger for the GOE.

Furthermore, the elemental sulfur produced *via* channel (1) from SO_2_ photochemistry might polymerize into S_2_, S_3_, S_4_, *etc.*, and end up contributing to insoluble S_8_-containing aerosols,^55^ the recycling of which could be responsible for the S-MIF signature in sedimentary rocks. If so, the tectonic reorganization, the abrupt rise of S-MIF signatures in sedimentary rocks and the “whiffs” of O_2_ in the late Archaean might be far from coincidental. Tectonics control volcanic eruptions, and the photochemistry of volcanic SO_2_ contributes to S-MIF and boosts the O_2_ budget.

O_2_ production from SO_2_ photochemistry could also be relevant in the contemporary atmospheres of other planets. For example, a layer of volcanic SO_2_ has been observed in the Venusian atmosphere, spanning heights from 48–65 km above the surface and with a measured maximal abundance of ∼130 ppm.^56^ More recently, O_2_ molecules in the a^1^Δ_g_ state have also been detected (through the O_2_(a^1^Δ_g_) dayglow) in the Venusian atmosphere.^57^ The present study provides unambiguous evidence for O_2_(a^1^Δ_g_) formation *via* SO_2_ photolysis at wavelengths around the H Lyman-α transition. Clearly, this is an O_2_ production mechanism that merits further scrutiny and, if necessary, incorporation into photochemical models for all planets with rich volcanically outgassed SO_2_.

## Data availability

The data supporting this study are available within the main text and the ESI.†

## Author contributions

K. Y. conceived the research. K. Y. and X. Y. designed the experiments and supervised the research. Y. C., Z. C., Z. L., Y. Z. and Z. L. performed the experiments. W. Q. Z., G. R. W., and X. M. Y. operated the FEL facility. Y. F., B. F., and D. Z. performed the theoretical calculations. Y. C., B. F., K. Y., M. N. R. A. and X. Y. wrote the manuscript. All authors discussed the results and commented on the manuscript.

## Conflicts of interest

There are no conflicts to declare.

## Supplementary Material

SC-014-D3SC03328G-s001
