# Supplementary material for: Vacuum ultraviolet photodissociation of sulfur dioxide and its implications for oxygen production in the early Earth's atmosphere
Source: Chem Sci. 2023 Aug 1;14(31):8255–61. doi: 10.1039/d3sc03328g (PMC10411858; doi:10.1039/d3sc03328g)
Supplement: SC-014-D3SC03328G-s001 [file SC-014-D3SC03328G-s001.pdf]

Electronic Supplementary Material (ESI) for Chemical Science.
This journal is © The Royal Society of Chemistry 2023

Supplementary Materials for

Vacuum ultraviolet photodissociation of sulfur dioxide and its implications for oxygen production in the early Earth's atmosphere

Yao Chang[1#], Yanlin Fu[1#], Zhichao Chen[1#], Zijie Luo[1,2], Yarui Zhao[1], Zhenxing Li[1], Weiqing Zhang[1], Guorong Wu[1], Bina Fu[1,4,5]*, Dong H. Zhang[1,5,6], Michael N.R. Ashfold[3], Xueming Yang[1,5,6]*, and Kaijun Yuan[1,4,5]*

1. *State Key Laboratory of Molecular Reaction Dynamics and Dalian Coherent Light Source, Dalian Institute of Chemical Physics, Chinese Academy of Sciences, 457 Zhongshan Road, Dalian, 116023, China*
2. *Marine Engineering College, Dalian Maritime University, Liaoning, 116026, China*
3. *School of Chemistry, University of Bristol, Bristol, BS8 1TS, UK*
4. *University of Chinese Academy of Sciences, Beijing 100049, P. R. China*
5. *Hefei National Laboratory, Hefei 230088, China*
6. *Department of Chemistry and Center for Advanced Light Source Research, College of Science, Southern University of Science and Technology, Shenzhen 518055, China*

[*] To whom correspondence should be addressed. E-mail addresses: kjyuan@dicp.ac.cn, bina@dicp.ac.cn, xmyang@dicp.ac.cn.

Supplementary texts

S1. Experimental Methods

The present experimental apparatus is equipped with two independently tunable vacuum ultraviolet (VUV) laser systems coupled with the time-sliced velocity map ion imaging (TS-VMI) detection scheme, as shown schematically in Fig. S1. The VUV-VUV TS-VMI apparatus has been described previously in detail.[1-3] Briefly, two independently tunable VUV laser beams have been generated by, respectively, the VUV free electron laser (FEL) and a sum-/difference-frequency four-wave mixing (FWM) scheme by a table-top laser system. Henceforth, these sources are denoted as VUV-FEL and VUV-FWM, respectively. The VUV-FEL, which runs in the high gain harmonic generation (HGHG) mode, was used to photoexcite SO_2 molecules to higher electronic states of interest. The typical pulse energy of the VUV FEL is >200 μJ (attenuated to ~ 10 μJ in the present experiments), with the duration of ~1.5 ps in the wavelength range $50 \leq \lambda \leq 150$ nm. The polarization of the VUV FEL was fixed in the horizontal plane and thus parallel to the front face of the MCP detector. The VUV-FWM beam at λ=130.092 nm was used to ionize the S(^{1}D) photofragment via the auto-ionization Rydberg state S*[$3s^2 3p^3(^2D^\circ)5s$ ($^1D^\circ_2$)]. The 130.092 nm light was generated as the difference-frequency ($2\omega_1 - \omega_2$) output in Kr gas, in which ω_1 was set at the frequency corresponding to a wavelength λ =212.556 nm and the ω_2 was scanned around λ ~580.654 nm so as to cover the Doppler profile. The ω_1 (λ =212.556 nm) radiation was produced by frequency doubling the output of a 355 nm (Nd:YAG laser, Spectra Physics Pro-290) pumped dye laser (Sirah, PESC-G-24, 3000 l/mm) operating at λ ~425 nm. Half of the 532 nm output of the same Nd:YAG laser was used to pump another dye laser (Sirah, PESC-G-24) which operated around λ ~580.654 nm (~8-10 mJ pulse energy). To avoid any unwanted secondary dissociations, an off-axis biconvex LiF lens was used to disperse the 212.556 nm and 580.654 nm outputs from the direction of 130.092 nm beam, and the 130.092 nm radiation remained defocused. The polarization vector of the VUV-FWM output was determined by the polarization of the 580.654 nm radiation, which was fixed in the horizontal direction.

The pulsed supersonic beam was generated by expanding a mixture of 1% SO_2

and Ar into the source chamber where it was skimmed before entering (through a 2 mm hole in the first electrode) and propagating along the centre axis of the ion optics assembly (IOA, 23-plate ion optics) mounted in the reaction chamber. The molecular beam was intersected at right angles by the photolysis and probe laser beams between the second and the third plates of the IOA. The SO_2 molecules were photodissociated by the VUV-FEL pulse, and the $S(^1D)$ photoproducts were subsequently probed by single photon excitation at λ = 130.092 nm. The resulting S^+ ions were accelerated through the remaining ion optics and detected by a dual microchannel plate (MCP) detector coupled with a P43 phosphor screen at the end of the 740 mm ion flight tube. In the current experiments, the detector was time gated to select ions with *m*/*z* 32 (15 ns gain pulse width). To confirm that the signal was from the intended two-color VUV pump-probe scheme, three images were recorded specifically under different experimental conditions: (i) with both pump (VUV-FEL) and probe (VUV-FWM) beams present in the interaction region; (ii) with the pump beam present but the probe beam being blocked, and (iii) with the pump beam blocked and the probe beam being present. The one-color photon induced background images recorded under conditions (ii) and (iii) were very weak and were subtracted from the two-color image recorded under condition (i). The speed of the S^+ product was calibrated by imaging the O^+ ions from the well-characterized multiphoton excitation and ionization of O_2 at 225.000 nm.[4,5]

S2. The Absorption Spectrum of SO_2

As shown in Fig. S2, the room temperature absorption spectrum of SO_2 displays extensive absorption at all UV wavelengths below ~400 nm.[6,7] The strong absorption band between 185 and 235 nm is associated with the transition from ground ($\tilde{X}^1A_1$) state to the $\tilde{C}^1B_2$ state ($\tilde{C}^1B_2 \leftarrow \tilde{X}^1A_1$). A weaker absorption band between 240-350 nm corresponds to the transition to the $\tilde{B}^1B_1$ state, which couples with the $\tilde{A}^1A_2$ state via a conical intersection.[8,9] The direct $\tilde{A}^1A_2 \leftarrow \tilde{X}^1A_1$ transition is electric dipole forbidden at C_{2v} geometries, and the $\tilde{A}$ state is expected to be a dark state (*i.e.*, not observed directly by optical spectroscopy). Another weak absorption band at λ >350 nm is ascribed to

the spin-forbidden transition to a triplet state ($\tilde{a}^3B_1 \leftarrow \tilde{X}^1A_1$), not shown in Fig. S2.

Photons of wavelength λ <219 nm have enough energy to break one of the SO bonds, leading to fragmentation,[10] whereas radiation at all longer wavelengths can only excite SO_2 to one of the excited states (the coupled $\tilde{B}^1B_1/\tilde{A}^1A_2$ states and/or the $\tilde{a}^3B_1$ state) which then decay radiatively.[11] Photodissociation of SO_2 via the $\tilde{C}^1B_2$ state has been investigated by several groups[12,13] due to its strong relation with the sulfur mass-independent fractionation (S-MIF). Since the $\tilde{C}^1B_2$ state correlates adiabatically to $SO(a^1\Delta) + O(^1D)$ products, the observed production of the lower energy $SO(X^3\Sigma^-)$ + $O(^3P)$ products must arise via non-adiabatic coupling. One possible pathway is internal conversion to the $\tilde{X}^1A_1$ state, which then dissociates to $SO(X^3\Sigma^-)$ and $O(^3P)$ products.[14] Alternatively, a conical intersection formed with the higher lying $\tilde{D}^1A_1$ state, which is repulsive and correlates with the $SO(X^3\Sigma^-) + O(^3P)$ asymptote, facilitates predissociation of SO_2 via the $\tilde{C}^1B_2$ state.[15] Another potential non-adiabatic pathway would involve intersystem crossing to the repulsive 2^3A_1 state, which correlates to the same triplet asymptote.[15]

The diffuse absorption bands at shorter wavelengths are assigned to transitions to Rydberg states. The $\tilde{E}$ state was observed by Vuskovic and Trajmar [16] by electron impact excitation and assigned to the absorption band centred at ~154 nm. Features identified at higher energy include the $\tilde{F}$ Rydberg state, with an origin at 134 nm,[17] and the $\tilde{G}$ and $\tilde{H}$ Rydberg states which can be reached at wavelengths ~120 nm.[18]

S3. Experimental Results at More VUV Wavelengths

Fig. S3 shows time-sliced ion images of the $S(^1D)$ photofragments recorded following photolysis of SO_2 at λ = 125.1, 130.1, 144.1 and 154.1 nm. The double headed red arrow in the figure shows the polarization direction of photolysis laser radiation. The well-resolved concentric rings evident in the images can be directly attributed to population of different vibrational states of the O_2 co-product in its ground ($X^3\Sigma_g^-$) or first excited ($a^1\Delta_g$) electronic state. Fig. S4 displays the corresponding $P(E_T)$ spectra, obtained by integrating over all recoil angles in the images shown in Fig. S3. The

superposed combs indicate the E_T values associated with formation of S(^{1}D) atoms together with O_2(X/a) products in different vibrational states. Best-fit simulations of these spectra and those shown in Fig. 2 return the O_2(X) and O_2(a) product vibrational state population distributions shown in Figs. S5 and S6.

S4. The Branching Ratio Measurements

Any quantitative assessment of the importance of SO_2 photolysis in the VUV region and its possible role in abiotic oxygen formation in the early Earth's atmosphere requires determination of the branching ratios of all dissociation channels. Since Lyman-α (121.6 nm) photons dominate the stellar VUV radiation field, we take this as the representative wavelength at which to measure the branching ratios. The energetically allowed channels from photodissociation of SO_2 at 121.6 nm are listed below,

$$SO_2 + h\nu \rightarrow SO(X^3\Sigma^-/a^1\Delta) + O(^3P) \qquad (I)$$

$$SO_2 + h\nu \rightarrow SO(X^3\Sigma^-/a^1\Delta) + O(^1D) \qquad (II)$$

$$SO_2 + h\nu \rightarrow SO(X^3\Sigma^-) + O(^1S) \qquad (III)$$

$$SO_2 + h\nu \rightarrow O_2(X^3\Sigma^-_g/a^1\Delta_g) + S(^3P) \qquad (IV)$$

$$SO_2 + h\nu \rightarrow O_2(X^3\Sigma^-_g/a^1\Delta_g) + S(^1D) \qquad (V)$$

$$SO_2 + h\nu \rightarrow O_2(X^3\Sigma^-_g/a^1\Delta_g) + S(^1S) \qquad (VI)$$

Of these, the fragmentation channels yielding O(^{1}D) + SO, O(^{1}S) + SO and S(^{1}D) + O_2 products have been detected using the VUV pump (at 121.6 nm) and VUV probe technique. Attempts to detect O(^{3}P), S(^{3}P) and S(^{1}S) products under similar experimental conditions yielded no detectable signals, suggesting that the O(^{3}P) + SO, S(^{3}P) + O_2 and S(^{1}S) + O_2 channels all have negligible quantum yield at this wavelength.

Three types of experiment were performed under similar experimental conditions: (a) SO_2 was excited at λ = 121.6 nm, and the O(^{1}D) product detected by one photon excitation at λ = 92.201 nm, via the (O*[$2s^22p^3(^2D^\circ_{3/2})3d$ ($^1F^\circ_3$)] $\leftarrow$ O(^{1}D)) transition and subsequent autoionization. The λ = 92.201 nm photons were generated by sum-frequency ($2\omega_1 + \omega_2$) FWM using Xe as the nonlinear medium, with ω_1 set at the

appropriate wavelength (λ = 222.568 nm) for two-photon resonant excitation of the Xe [$5p^5(^2P^\circ_{1/2})6p \leftarrow 5p^6\ ^1S_0$] transition and ω_2 fixed at λ = 537.671 nm; (b) SO_2 was excited at λ = 121.765 nm and the O(^{1}S) product was then ionized by absorbing another 121.765 nm photon via the autoionizing O*[$2s^22p^3(^2P^\circ)3s\ (^1P^\circ)$] $\leftarrow$ O(^{1}S) transition; (c) SO_2 was excited at λ = 121.895 nm, and the S(^{1}D) product was then ionized by absorbing another 121.895 nm photon via the autoionizing S*[$3s^23p^3(^2D^\circ)4d\ (^1D^\circ)$] $\leftarrow$ S(^{1}D) transition. The λ = 121.6, 121.765 and 121.895 nm photons were each generated by difference-frequency ($2\omega_1 - \omega_2$) FWM using two λ = 212.556 nm photons and one photon at λ ~840 nm in Kr gas. Though the photolysis wavelengths applied in these experiments are not identical, it is rational that the parent SO_2 cross sections and dissociation dynamics are the same (to within the quoted error bars). [The cross sections for SO_2 absorption at λ = 121.6, 121.765 and 121.895 nm are, respectively, 3.04×10^{-17}, 3.11×10^{-17} and 3.16×10^{-17} cm^2 (ref. 4)]. Thus, the branching ratios of the O(^{1}D) + SO, O(^{1}S) + SO and S(^{1}D) + O_2 channels can be deduced by calibrating the detection efficiencies of processes (a)-(c) together with a proper consideration of the Doppler broadening effects accompanying each measurement.

Calibration of the detection efficiencies. To calibrate the detection efficiencies, we need to ensure that the pump and probe VUV beams in the above experiments are overlapped exactly in time and space, and to consider the powers of the VUV beams and the transition probabilities for the O(^{1}D), O(^{1}S) and S(^{1}D) fragment detection schemes. The spot size of the VUV beams generated by sum/difference-frequency FWM can be tuned by the lens which focuses the ω_1 and ω_2 beams into the mixing cell. Though different VUV beams were used in experiments (a)-(c), arranging that these beams all had the same size in the interaction region ensured that the measurement error associated with their spatial overlap was minimal. To visualize the spot size of the VUV beams, NO^+ ion images were recorded following single VUV photoionization of NO molecules (seeded as a 1% mixture in He carrier gas) with the ion optics defocussed, as shown in Fig. S7A. The horizontal streak in these images is caused by the VUV beam crossing the NO molecular beam and ionizing the NO along the VUV beam propagation direction, while the vertical width is proportional to the spot size of the VUV beam

(confirmed by simulation of the relationship between the NO^+ ion images and the initial photo-ion profile in the intersection region, as shown in Fig. S7C). The normalized intensity vs pixel profiles along the vertical axis of the NO^+ ion images obtained using the different VUV photoionization schemes (Fig. S7B) demonstrate the very similar spot sizes of all VUV-FWM beams, ensuring that the same conditions of spatial overlap between the pump and probe VUV beams were achieved in experiments (a)-(c). Temporal overlap of the pump and probe VUV beams was easily achieved using a digital delay generator (DG645).

The next important issue is to calibrate the powers of the pump and probe VUV beams. The integrated ion signals shown in Fig. S7A allowed determination of the relative powers of the VUV beams at λ = 92.201, 121.6, 121.765, and 121.895 nm – namely, 0.017: 1.000: 0.927: 0.317 (assuming relative NO photoionization efficiencies at these wavelengths of, respectively, 36.16, 4.84, 4.89 and 4.97 [19]). Given (i) that the photofragment detection efficiencies are proportional to the power of the probe laser and (ii) the $O^*[2s^22p^3(^2D^\circ_{3/2})3d\ (^1F^\circ_3)] \leftarrow O(^1D_2)$, $O^*[2s^22p^3(^2P^\circ)3s\ (^1P^\circ_1)] \leftarrow O(^1S_0)$ and $S^*[3s^23p^3(^2D^\circ)4d\ (^1D^\circ_2)] \leftarrow S(^1D_2)$ transition probabilities (1.230×10^8, 2.060×10^8 and 0.455×10^8 s^{-1}, respectively [20,21]), the calibrated relative detection efficiencies in experiments (a)-(c)) were determined as $O(^1D)$: $O(^1S)$: $S(^1D)$ = 0.011: 1.000: 0.076.

Calibration for the Doppler effect. When measuring ion images such as those reported in Figs. 1 and S3 it is essential to scan the detection laser wavelength across the product Doppler width, since the large recoil speeds can translate into very large Doppler widths and correspondingly reduced sensitivity. The present branching ratio determinations were made using fixed probe wavelengths. To calibrate for Doppler effects in the detection of the $O(^1D)$, $O(^1S)$ and $S(^1D)$ fragments of current interest, $O(^1D)$, $O(^1S)$ and $S(^1D)$ fragment images were first recorded by accumulating for a known number of laser shots whilst holding the detection laser at its chosen fixed wavelength. These images are shown in Figs. S8A-C**.** The integrated signal counts for the $O(^1D)$, $O(^1S)$ and $S(^1D)$ fragments were, respectively, 2.67×10^5, 3.88×10^5 and 9.64×10^5. Images for these same three atom products were also recorded whilst repeatedly scanning the VUV detection laser across the full product Doppler width.

These images are shown in Figs. S8D-F. Clearly, the images recorded without Doppler scanning show only a part of the images obtained with Doppler scanning. The integrated signal profiles along the horizontal pixel range in these images are shown in Figs. S8G-I. Normalizing these profiles allowed determination of the following species-specific signal strength ratios without vs with Doppler scanning: 0.21, 0.70 and 0.87 for $O(^1D)$, $O(^1S)$ and $S(^1D)$, respectively.

The last element required for determining the product branching ratios listed in Table S1 are the respective powers of the pump VUV laser used in the calibration experiments (which for the $O(^1D)$, $O(^1S)$ and $S(^1D)$ calibration experiments detailed here were in the ratio 1.000: 0.927: 0.317, respectively). Combining the respective measured signal counts, detection efficiencies, Doppler corrections and pump powers results in the following calibrated product channel branching ratios: $O(^1D)$ + SO: $O(^1S)$ + SO: $S(^1D)$ + O_2 = 0.710: 0.004: 0.286. The results of two further branching ratio determinations using the above methods are also shown in Table S1. Thus, we estimate a branching ratio of 30 ± 5 % for the $S(^1D) + O_2(X^3\Sigma^-_g/a^1\Delta_g)$ channel in SO_2 photolysis at 121.6 nm. The overall error associated with these experimental results was estimated to be ~15%.

S5. Computational Details

The present work employed the explicitly correlated version (F12) of the internally contracted multi-reference configuration interaction (ic-MRCI-F12) method, together with Dunning's augmented correlation-consistent triple-zeta (aug-cc-pVTZ) basis set, to calculate potential energy curves relevant to SO_2 photodissociation. In some cases, the Davidson correction (+Q) was also employed for the MRCI-F12, to better account for contributions from higher excitations; these results are denoted as ic-MRCI-F12+Q. The electronic structure calculations were performed using the MOLPRO 2018 suite of programs.[22] The reference wave functions were based on state averaged complete active space self-consistent field (SA-CASSCF) wavefunctions and the full-valence active space employed consisted of 18 electrons in 12 active orbitals (i.e. an 18(12) active space, with the 1s orbitals of O and S and 2s and 2p orbitals of S frozen). Potential

energies were calculated in C_s symmetry, and the SA-CASSCF wavefunctions were averaged over the six lowest energy singlet and six lowest energy triplet states of both A' and A'' symmetry, i.e. a total of twelve A' and twelve A'' states were finally obtained.

Table S2 compares the equilibrium geometry of the global minimum of the ground state of SO_2 calculated in the present work with available experimental data [23-25] and with previous theoretical results.[8,14] The current theoretical results are in very good agreement with the recent experimental results and confirm the C_{2v} equilibrium geometry of the ground state.[23,24] To obtain the correct asymptotic energies for different S + O_2 product pairs (i.e. dissociation energies for the various electronic states), the MRCI-F12+Q energies of S and O_2 were calculated separately, using 12(8) and 6(5) active spaces for O_2 and S, respectively, in both the CASSCF/AVTZ and MRCI-F12+Q/AVTZ calculations. Table S3 shows that the dissociation energies derived here accord well with the experimental values.

Fig. S9 shows the calculated vertical excitation energies of the first six singlet and triplet spin states of both A'' and A' symmetry, and their diabatic correlations with various SO + O and S + O_2 product pairs. This figure serves to illustrate (just some of) the many A' and A'' states that correlate with these various low energy dissociation limits (and we note that two excited states with quintet spin multiplicity will also correlate with the ground state products). The density of electronic states in the vertical Franck-Condon region in the energy range of current interest (8-10 eV) will be very high, which can be expected to facilitate easy transfer of population between states (via internal conversions and/or intersystem crossings) once SO_2 is excited to these states.

Table S1. The branching ratios of detectable dissociation channels for SO_2 photodissociation at 121.6 nm determined in three separate experiments.

Measurement	The branching ratios of dissociation channels		
	$O(^1D) + SO(X^3\Sigma^-)$	$O(^1S) + SO(X^3\Sigma^-)$	$S(^1D) + O_2(X^3\Sigma^-{}_g)$
1st	0.710	0.004	0.286
2nd	0.725	0.004	0.271
3rd	0.677	0.003	0.320

Table S2. Comparisons of the geometry of the global minimum of the ground state of SO_2 calculated in the present work, cf. values obtained in previous theoretical and experimental studies ($R_{S\text{-}O}$ in Å and ϕ in degrees).

	$R_{S\text{-}O}$	$R_{S\text{-}O}$	ϕ_{oso}
MRCI-F12/AVTZ[a]	1.434	1.434	119.7
CCSD-F12/AVTZ[a]	1.435	1.435	119.2
CCSD /AVQZ[b]	1.443	1.443	119.3
MRCI /AVTZ[c]	1.454	1.454	119.2
Exp.[d]	1.432	1.432	119.5
Exp.[e]	1.432	1.432	119.5
Exp.[f]	1.431	1.431	119.3

[a] this work, [b] Ref. [14]; [c] Ref. [8]; [d] Ref. [23]; [e] Ref. [24]; [f] Ref. [25].

Table S3. Comparisons of the dissociation energies of SO_2 (in eV) to S + O_2 products in various electronic states, defined relative to the global minimum of the ground state, from the present and selected previous studies.

S + O_2	$S(^3P) + O_2(^3\Sigma^-{}_g)$	$S(^3P) + O_2(^1\Delta_g)$	$S(^1D) + O_2(^3\Sigma^-{}_g)$	$S(^1D) + O_2(^1\Delta_g)$
MRCI-F12/AVTZ[a]	5.81	6.73	6.96	7.88
Exp.[b]	5.9	6.9	7.0	8.0
Exp.[c]	5.7±0.1	6.68	6.84	7.82

[a]this work; [b]Ref. [26] [c]Ref. [27].

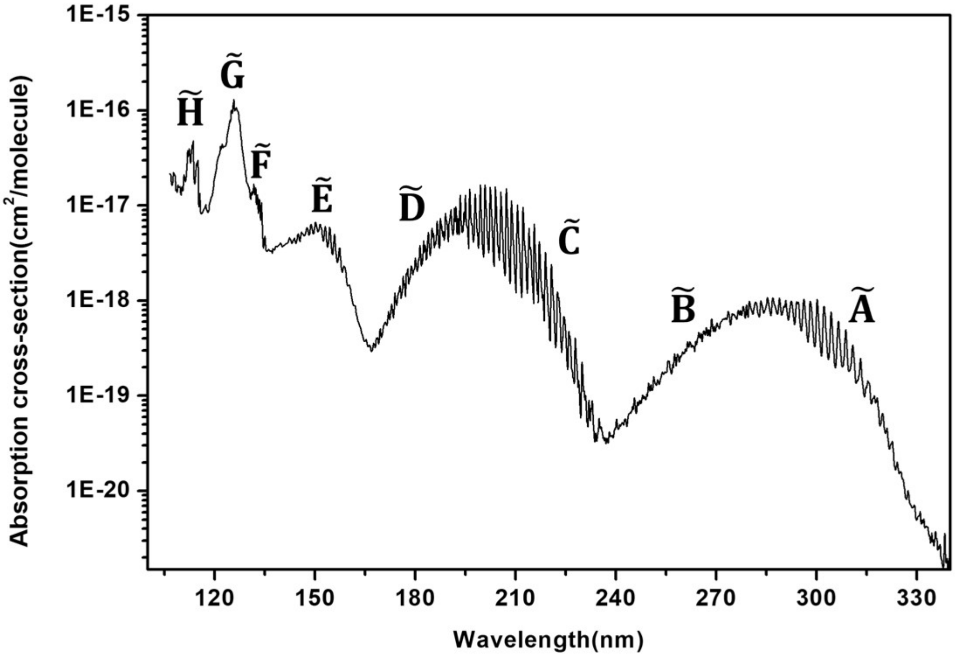

Fig. S1. The absorption spectrum of SO_2 based upon the spectra of Golomb *et al.*[6] (106 – 217 nm) and Danielache *et al.*[7] (217 – 340 nm). The regions traditionally associated with different excited states summarized in the Introduction are labelled for convenience.

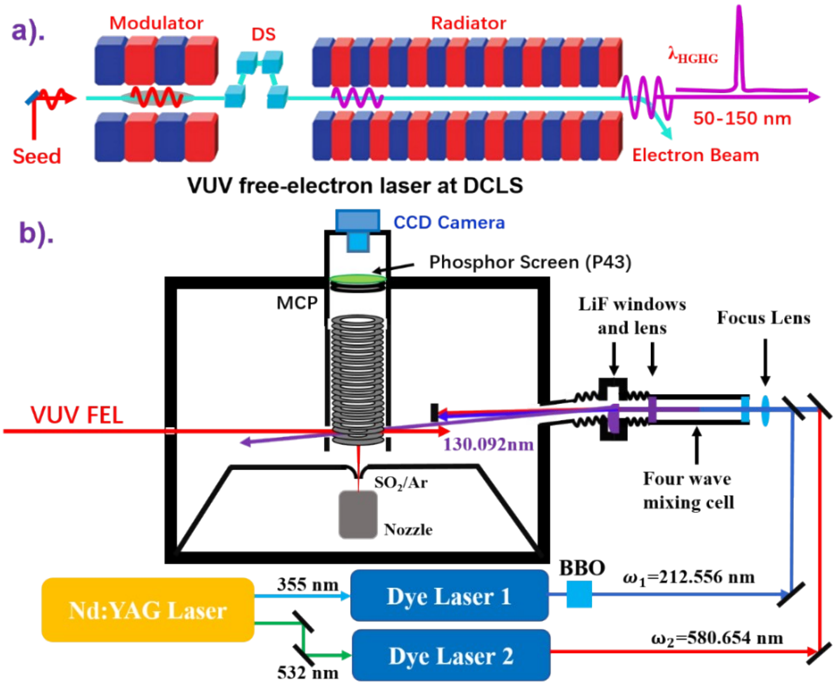

Fig. S2. a) Schematic of the VUV free electron laser (VUV FEL) beam line at the Dalian Coherent Light Source (DCLS). b) The arrangement of VUV-pump and VUV-probe time-sliced velocity-map imaging (TS-VMI) system for SO_2 photochemistry. The tunable VUV pump source (λ_1~120-160 nm) comes from the VUV FEL beam line, and the fixed VUV probe light (λ_2=130.092 nm) is generated using a two-photon resonance-enhanced four-wave mixing scheme with the fundamental radiations dispersed from the propagation direction of the VUV radiation. DS: dispersion section; MCP: micro-channel plate; BBO: beta barium borate crystal.

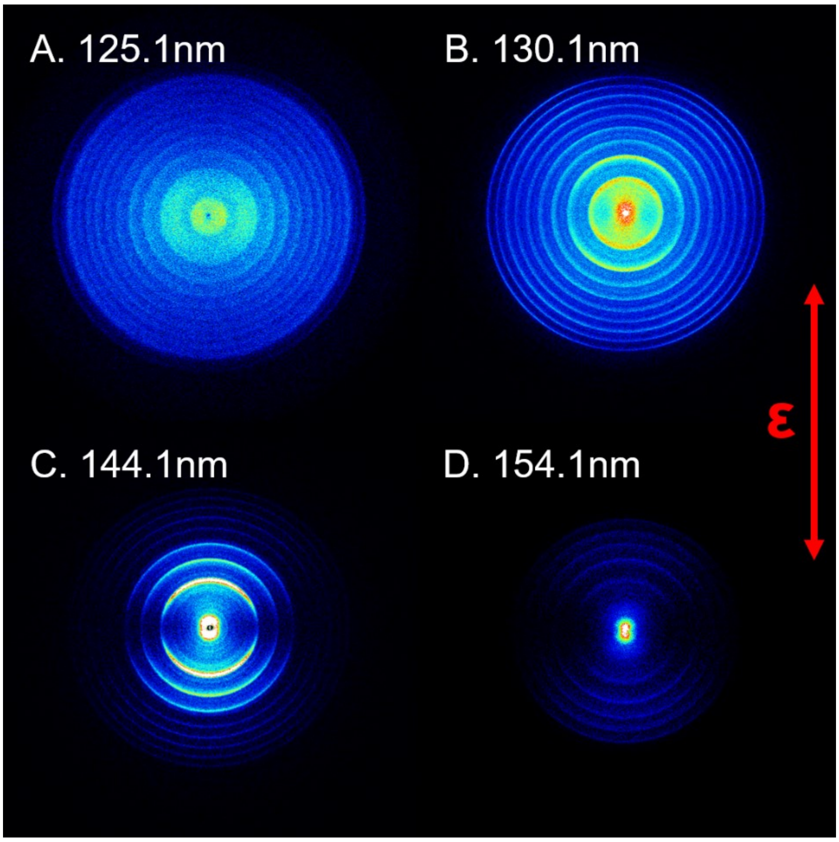

Fig. S3 Time-sliced images of the S(^{1}D) products from SO_2 photodissociation. These images were recorded at photolysis wavelengths of: A) 125.1 nm, B) 130.1 nm, C) 144.1 nm and D) 154.1 nm. The double headed red arrow shows the polarization direction of the photolysis radiation. The ring features correspond to the population of different vibrational states of the partner O_2(X/a, *v*) products.

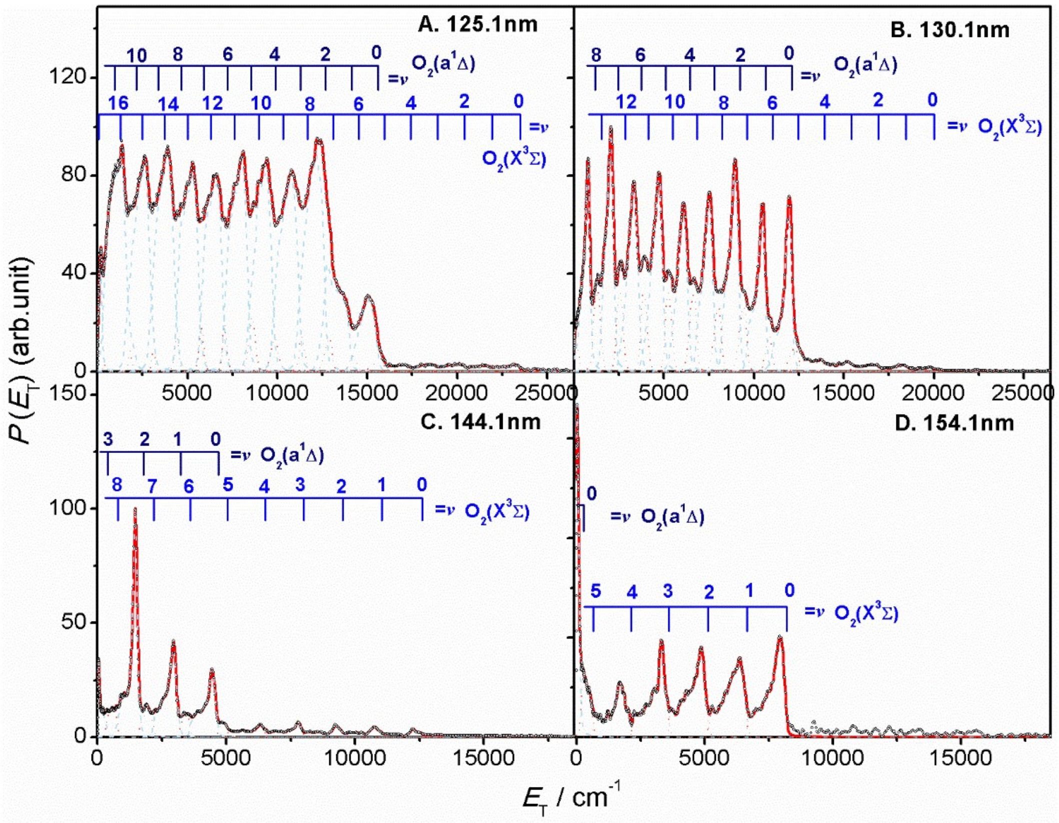

Fig. S4. Product total kinetic energy distribution ($P(E_T)$) spectra derived from images taken at A) 125.1 nm, B) 130.1 nm, C) 144.1 nm and D) 154.1 nm (shown in Fig. S3), in red, along with the best-fit simulations of these spectra, in cyan dashed lines and orange dotted lines. The superposed combs indicate the E_T values associated with formation of the various vibrational states of O_2(X/a).

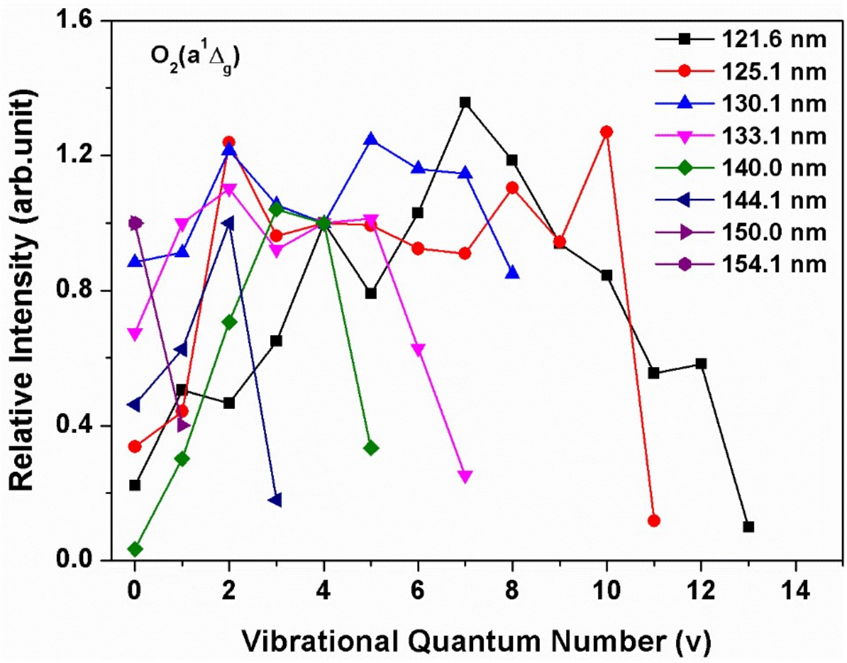

Fig. S5. Relative populations of different vibrational states of the O_2(a) products formed via the S(^{1}D) + O_2(a) channel derived from analysis of S(^{1}D) images recorded at eight photolysis wavelengths.

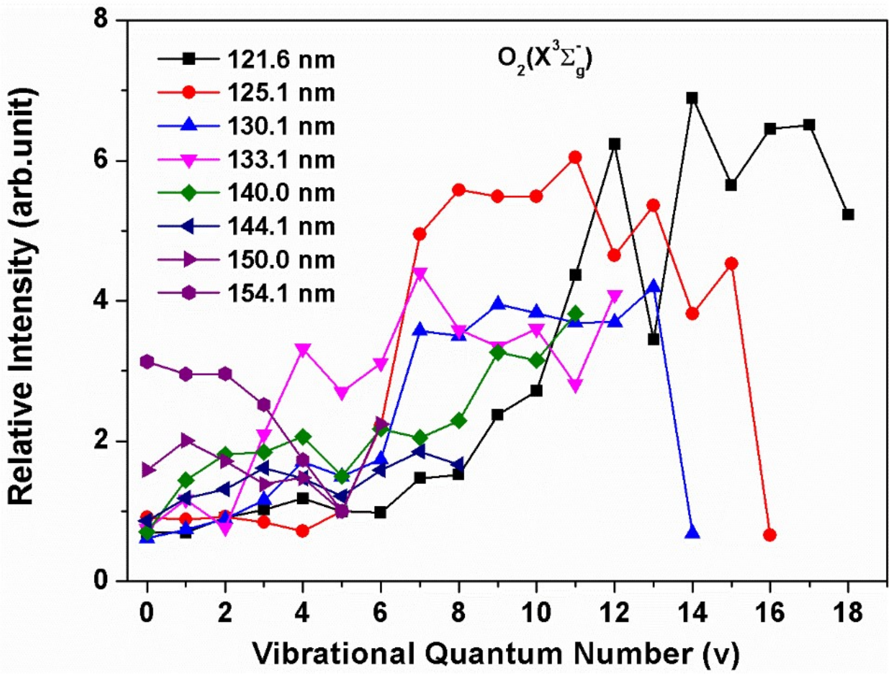

Fig. S6. Relative populations of different vibrational states of the O_2(X) products formed via the S(^{1}D) + O_2(X) channel derived from analysis of S(^{1}D) images recorded at eight photolysis wavelengths.

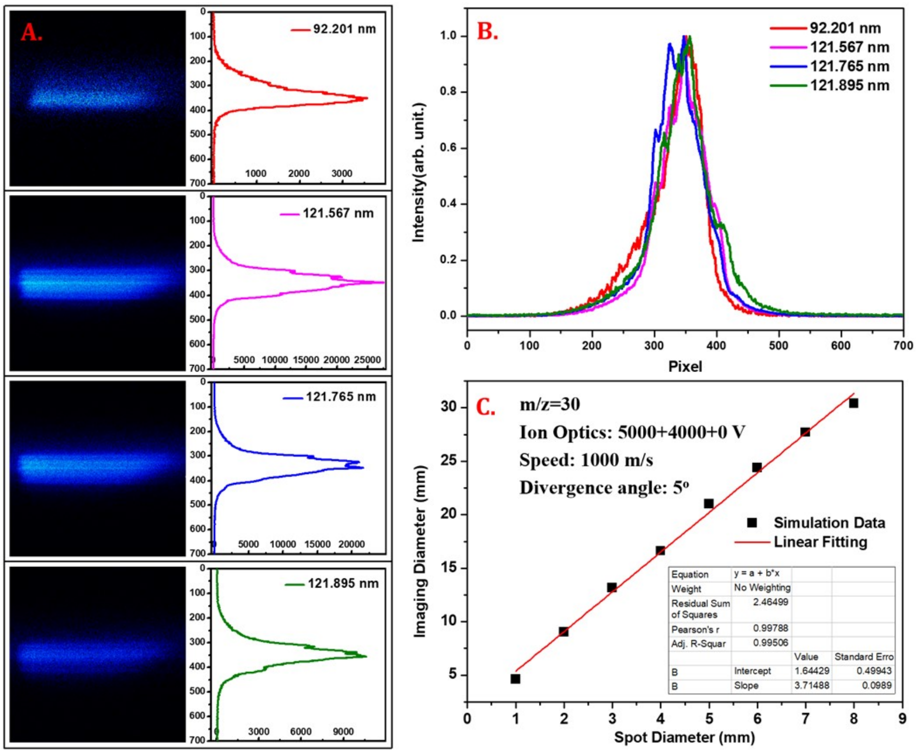

Fig. S7. A) Velocity-map images of NO^+ ions in the NO (1% in He) molecular beam along with the corresponding signal profiles (intensity vs pixel plots) at wavelengths λ = 92.201 nm, 121.6 nm, 121.765 nm and 121.895 nm (from top to bottom, respectively). B) Normalized NO^+ signal profiles obtained using the various VUV laser sources, demonstrating the very similar spatial profiles of the NO^+ images produced by the different VUV lasers. C) Simulated diameters of NO^+ images (obtained using the Simion 8.0 software) as a function of the diameter of the nascent NO^+ at the reaction center, where it has been assumed that the volume containing the nascent NO^+ ions is determined by the spot size of the VUV light. The simulation displays a linear correlation between the NO^+ image size and the spot size of the VUV beams. These experiments confirm that each VUV beam had the same spot size in the interaction region.

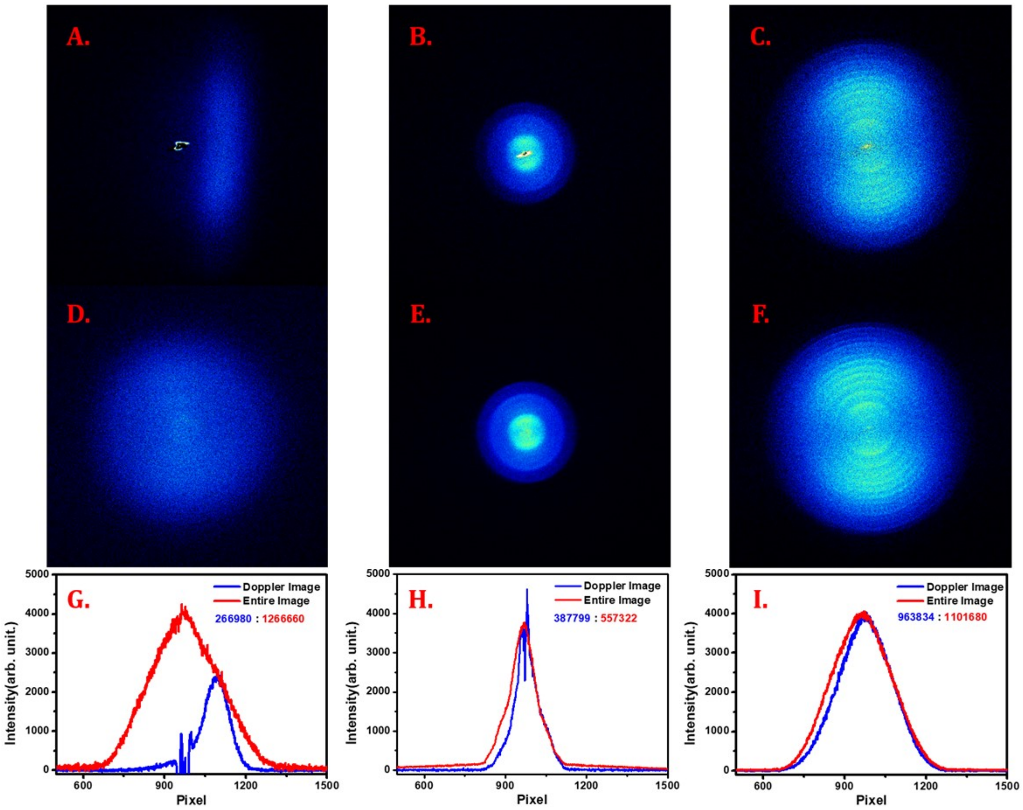

Fig. S8. Experimental results from the photodissociation of SO_2 at the wavelengths λ = 121.6 nm, for the $O(^1D)$ + SO channel (A, D, G); 121.765 nm, for the $O(^1S)$ + SO channel (B, E, H); and 121.895 nm, for the $S(^1D)$ + O_2 channel (C, F, I). (A-C): Recorded ion images accumulating over the same number of laser shots for $O(^1D)$, $O(^1S)$ and $S(^1D)$ fragment detection without scanning the probe laser wavelength. (D-F): Ion images for these same three atomic products recorded with the probe laser wavelength scanned so as to cover the entire product Doppler width. (G-I): Integrated signal profiles along the horizontal pixel scale of the respective images.

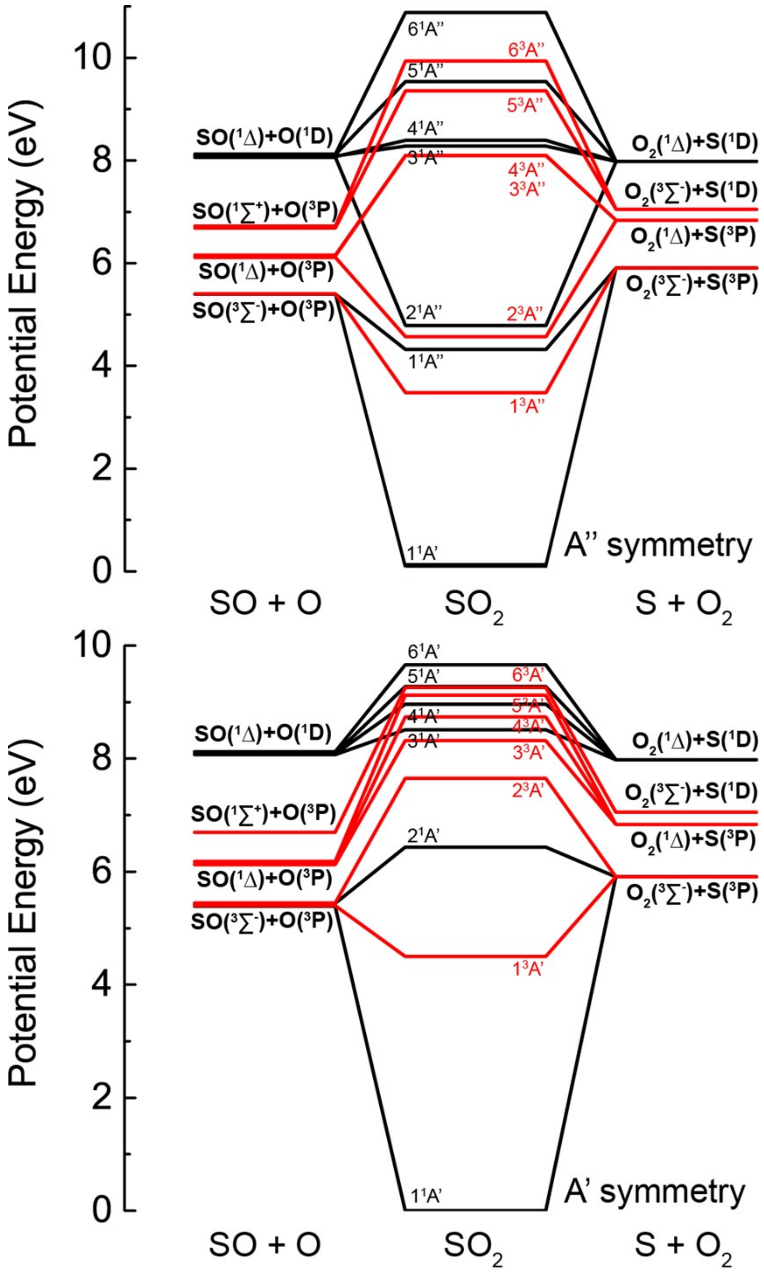

Fig. S9. Calculated vertical excitation energies of the singlet (black) and triplet (red) states of A'' symmetry (upper panel) and of A' symmetry (lower panel), along with their diabatic correlations with selected low energy SO + O and S + O_2 dissociation limits.

References

1 J. Zhou, Y. Zhao, C. S. Hansen, J. Yang, Y. Chang, Y. Yu, G. Cheng, Z. Chen, Z. He, S. Yu, H. Ding, W. Zhang, G. Wu, D. Dai, C. M. Western, M. N. R. Ashfold, K. Yuan and X. Yang, *Nat. Commun.*, 2020, **11**, 1547.

2 Y. Chang, S. Yu, Q. Li, Y. Yu, H. Wang, S. Su, Z. Chen, L. Che, X. Wang, W. Zhang, D. Dai, G. Wu, K. Yuan and X. Yang, *Rev. Sci. Instrum.*, 2018, **89**, 063113.

3 Z. Li, M. Zhao, T. Xie, Z. Luo, Y. Chang, G. Cheng, J. Yang, Z. Chen, W. Zhang, G. Wu, X. Wang, K. Yuan and X. Yang, *J. Phys. Chem. Lett.*, 2021, **12**, 844-849.

4 A. T. J. B. Eppink and D. H. Parker, *Rev. Sci. Instrum.*, 1997, **68**, 3477-3484.

5 D. H. Parker and A. T. J. B. Eppink, *J. Chem. Phys.*, 1997, **107**, 2357-2362.

6 D. Golomb, K. Watanabe and F. F. Marmo, *J. Chem. Phys.*, 1962, **36**, 958-960.

7 S. O. Danielache, C. Eskebjerg, M. S. Johnson, Y. Ueno and N. Yoshida, *J. Geophys. Res.*, 2008, **113**, D17314.

8 C. Xie, X. Hu, L. Zhou, D. Xie and H. Guo, *J. Chem. Phys.*, 2013, **139**, 014305.

9 C. Leveque, A. Komainda, R. Taieb and H. Koppel, *J. Chem. Phys.*, 2013, **138**, 044320.

10 S. Becker, C. Braatz, J. Lindner and E. Tiemann, *Chem. Phys.*, 1995, **196**, 275-291.

11 H. Okabe, *J. Am. Chem. Soc.*, 1971, **93**, 7095-7096.

12 C. Xie, B. Jiang, J. Klos, P. Kumar, M. H. Alexander, B. Poirier and H. Guo, *J. Phys. Chem. A*, 2017, **121**, 4930-4938.

13 B. R. Cosofret, S. M. Dylewski and P. L. Houston, *J. Phys. Chem. A*, 2000, **104**, 10240-10246.

14 J. Klos, M. H. Alexander, P. Kumar, B. Poirier, B. Jiang and H. Guo, *J. Chem. Phys.*, 2016, **144**, 174301.

15 H. Katagiri, T. Sako, A. Hishikawa, T. Yazaki, K. Onda, K. Yamanouchi and K. Yoshino, *J. Mol. Struct.*, 1997, **413-414**, 589-614.

16 L. Vušković and S. Trajmar, *J. Chem. Phys.*, 1982, **77**, 5436-5440.

17 V. Y. Foo, C. E. Brion and J. B. Hasted, *Proc. Roy. Soc. Lond. A*, 1971, **322**, 535-554.

18 M. Suto, R. L. Day and L. C. Lee, *J. Phys. B: At. Mol. Phys.*, 1982, **15**, 4165-4174.

19 R. M. Reese and H. M. Rosenstock, *J. Chem. Phys.*, 1966, **44**, 2007-2009.

20 N. C. Deb and A. Hibbert, *At. Data Nucl. Data Tables*, 2008, **94**, 561-602.

21 K. Butler and C. J. Zeippen, *J. Phys. IV*, 1991, **01**, C1-141-152.

22 H. J. Werner, P. J. Knowles, R. Lindh, F. R. Manby and M. Schütz,

23 G. Herzberg. *Molecular Spectra and Molecular Structure (III): Electronic Spectra and Electronic Structure of Polyatomic Molecules*. Princeton: Van Nostrand, 1966, 745.

24 S. Mai, P. Marquetand and L. Gonzalez, *J. Chem. Phys.*, 2014, **140**, 204302.

25 Y. Morino, Y. Kikuchi, S. Saito and E. Hirota, *J. Mol. Spectrosc.*, 1964, **13**, 95-118.

26 C. S. Effenhauser, P. Felder and J. R. Huber, *Chem. Phys.*, 1990, **142**, 311-320.

27 C. Lalo and C. Vermeil, *J. Photochem.*, 1975, **3**, 441-454.
